# Prolonged patent ductus arteriosus exposure and risk for late acute kidney injury in extremely preterm infants

**DOI:** 10.1038/s41372-026-02566-4

**Published:** 2026-02-05

**Authors:** Kelly Muterspaw, Russel Griffin, David Askenazi, Samuel J Gentle

**Affiliations:** 1 Department of Pediatrics, University of Alabama at Birmingham, Birmingham, AL USA; 2 Department of Epidemiology, University of Alabama at Birmingham, Birmingham, AL USA; 3 Department of Pediatrics, Yale School of Medicine, New Haven, CT USA

**Keywords:** Risk factors, Acute kidney injury, Congenital heart defects

## Abstract

**Objective:**

To study duration of hemodynamically significant patent ductus arteriosus (HPDA) exposure increases the risk of late acute kidney injury (AKI) and severity of AKI.

**Study design:**

This was a single-center retrospective cohort study. Included infants born between 22 and 28.6 weeks’ gestation with >1 echocardiographic finding for HPDA were stratified by HPDA duration: 4–7 weeks, 8–11 weeks, and greater than 12 weeks. AKI was determined utilizing KDIGO AKI criteria. Logistic regression analysis was used to evaluate odds ratios of each HPDA exposure groups for any AKI and severe (stage 2 or 3) AKI.

**Results:**

Among the 216 infants, 39(18%) developed AKI and 27(13%) developed severe AKI. Infants exposed to ≥12 weeks of HPDA exposure had a 3.96 (95% CI 1.32–11.87) higher odds of AKI, which was nonsignificant after adjustment for gestational age (aOR 2.37; 95% CI 0.72–7.78).

**Conclusion:**

Whether longer HPDA exposure increases risk for AKI development requires further investigation from trials of late PDA closure.

## Introduction

At least one of three infants born at less than 28 weeks’ gestation develops acute kidney injury (AKI). Neonatal AKI is a multi-faceted condition that can be caused by decreased renal perfusion, ischemia, nephrotoxins, and vascular abnormalities [1]. AKI in extremely preterm infants are associated with increased morbidity and mortality, with a 3-fold increased odds of death [2]. AKI has been associated with a hemodynamically-significant patent ductus arteriosus (HPDA) which is due to shunting of blood away from abdominal organs and resulting in poor renal perfusion and/or the medications used to close the PDA [3, 4]. Recent randomized trials of early pharmacologic closure have not demonstrated a reduction in short term adverse clinical outcomes of intraventricular hemorrhage, necrotizing enterocolitis, bronchopulmonary dysplasia, and death, however AKI was not reported [5, 6].

In many programs expectant management of PDAs has become an increasingly common approach and will likely continue following the publication of these trials. Infants may therefore endure longer durations of PDA exposure, which may increase infants’ risk of late onset AKIs. Associations between the duration of hypotension and renal hypoperfusion with AKI have been previously reported in other clinical contexts such as hypoxic ischemic encephalopathy and congenital heart disease [7, 8]. Our group has recently published observational data showing the association of the duration of PDA exposure with the development of BPD associated pulmonary hypertension (BPD-PH) [9, 10]. However, the relationship between PDA exposure duration and the associations with late AKI has been understudied.

We conducted a single-center cohort study in infants born <29 weeks’ gestation to evaluate this potential association between the duration of PDA exposure and late AKI risk. We hypothesized that the presence and duration of HPDA would be associated with increased prevalence and severity of late AKI following adjustment for known covariates known to increase risk for late AKI.

## Methods

### Ethics approval and consent to participate

The Institutional Review Board (IRB) at the University of Alabama at Birmingham reviewed the study and determined that it was exempt from ongoing review, as it involved the secondary use of identifiable private information. All methods were performed in accordance with the relevant guidelines and regulations. In accordance with the IRB’s determination, informed consent for participation was not required.

### Study design and patient population

Data for this observational cohort study was collected by retrospective chart review of infants born between 2017 and 2022 at the University of Alabama Birmingham. The institutional review board at the University of Alabama at Birmingham deemed the investigation exempt (as it only involved secondary use of identifiable private information). Infants were included in the study if they were born at gestational ages between 22 and 28.6 weeks, received at least one echocardiogram, and requiring respiratory support at 28 days of life. The latter was included because all infants at our center on respiratory support at 28 postnatal days receive an echocardiogram for evaluation of pulmonary hypertension. Infants then receive follow-up echocardiogram evaluations monthly until discharge, thus providing a cohort in which the duration of PDA exposure could be estimated. Infants were excluded if death occurred prior to 28 postnatal days, no echocardiogram was performed during the hospitalization, or major congenital anomalies were present (including structural congenital heart disease).

### Study exposures

The presence and persistence of PDA were evaluated in three dimensions: (1) as a binary outcome (yes/no), (2) hemodynamic significance (yes/no), and (3) strata of PDA exposure duration. For binary exposure classifications, we included echocardiograms between postnatal day 28 (the day in which systematic echocardiograms were performed in our patient population) and discharge. Hemodynamic significance was determined by clinical and echocardiographic observations as follows: echocardiographic criteria included PDA diameter greater than 1.5 mm and one of the following: a) ductal systolic flow velocity <2.8 m/s b) reverse diastolic flow in the descending aorta or c) ratio of left atrium to aortic root >1.6 [11–13].

### Study outcomes

Outcomes included late AKI which occurred at ≥4 weeks postnatal to match the PDA ascertainment in this study. AKI was defined by Kidney Disease Improving Global Outcomes Criteria using changes in serum creatinine only. Stage 1 was defined as 1.5–1.9 times baseline OR ≥ 0.3 mg/dL from baseline. Stage 2 was defined 2.0–2.9 times baseline creatinine. Stage 3 was 3.0 times baseline OR ≥ 2.5 0 mg/dL. Baseline creatine was determined as the lowest measured creatinine after 28 days of life. This creatinine value was chosen to establish a more proximal association between the duration of PDA exposure beyond this postnatal age and risk for late AKI.

### Demographic variables and other covariates

The demographic variables collected are used to evaluate associations between infant’s late AKI and severity of late AKI in relation to baseline characteristics. These included gestational age in weeks, birth weight in grams, multiple gestation, sex, race, antenatal steroids, Caesarean section delivery, histological chorioamnionitis, small for gestational age, and prolonged rupture of membranes (>18 h). Clinical characteristics were also collected to evaluate differences in AKI groups. Clinical characteristics included bronchopulmonary dysplasia (BPD) by severity [14], PDA, moderate to large PDA, pharmacologic treatment of PDA, grade 3 or 4 intracranial hemorrhage, early onset sepsis (bacteremia at <72 h after birth), late onset sepsis (bacteremia occurring at >72 after birth), severe retinopathy of prematurity, necrotizing enterocolitis (NEC) greater than or equal to stage 2, pulmonary hypertension, and death.

### Sample size

In prior cohort studies, the risk for AKI in infants <29 weeks’ gestation (as included in this investigation) is up to 48% [2]. Additionally, investigations relating risk of PDA and AKI have reported an odds ratio of 3.7 for AKI in infants with a PDA as compared to infants without a PDA [4]. The proportion of infants with a PDA in this population had been previously reported [10] to be around 30% of infants [10]. Based on these data, to achieve 80% power using an alpha value of 0.05, 155 infants would be needed to related PDA to late AKI in this patient population. Given the anticipated correlation between gestational age and PDA for which adjustment was anticipated as well as the additional need to evaluate the duration of PDA exposure with AKI, the sample size was further inflated to 200 patients.

### Statistics

Baseline demographics and clinical characteristics were compared by AKI groups. Comparison groups included late AKI versus no late AKI and analyzing late severe AKI (stage 2 or 3) versus stage 1 AKI and no late AKI. Categorical data was evaluated using chi square analysis. Continuous measures were tested for normality followed by appropriate parametric or nonparametric post-tests.

Logistic regression analysis was performed to assess the associations between the presence of a HPDA and the outcomes of interest (late AKI and severe late AKI). Two PDA exposures were assessed: HPDA as a binary exposure and duration of HPDA using strata of exposure duration: 4–7 weeks, 8–11 weeks and ≥12 weeks. Infants’ strata classification was defined by the latest echocardiogram demonstrating the presence of a PDA such that each infant could only be in one stratum (e.g. an infant exposed to a PDA at ≥12 weeks was not included in the 8–11 weeks stratum). The frequency of screening echocardiograms (monthly) precluded more precise estimates of the duration of ductal exposure. Therefore, as the duration of exposures were intrinsically estimates, we similarly characterized the duration of exposure with the same interval.

The adjusted model incorporated unique demographic variables that differed between AKI groups (*p* < 0.05). Results are reported as OR with 95% confidence interval (Cl). A *p* value of <0.05 was considered significant. Statistical analyses were performed using IBM^®^ SPSS^®^ Statistics Version: 28.0.1.1.

## Results

### Patient population

There were 398 infants born at the time of evaluation with gestational ages. Of the 398 infants born between 22 and 28.6 weeks gestational age, 182 (46%) did not require prolonged respiratory support or did not receive an echocardiographic evaluation; thus, 216 (54%) infants were available for analysis. Late AKI occurred in 39/216 (18%). Of infants with late AKI, 12/39 (31%) had stage 1 AKI and 27/39 (69%) had late severe AKI.

### Demographic characteristics

Demographics evaluated included, gestational age, birth weight, multiple gestations, sex, race, antenatal steroids, delivery via cesarean section, histologic chorioamnionitis, small for gestational age, and prolonged rupture of membranes. The gestational age (median; IQR) of those with late AKI (24.1; 23.1–26.1) and severe late AKI (23.9; 23.1–25.9) was lower than infants without late AKI (25.9; 24.3–27.4). Additionally, more infants with late (49%; *p* < 0.001) and severe late AKI (52%; *p* < 0.001) were born at <24 weeks’ gestation compared to infants without late AKI (16%). Similarly, the birth weights (median; IQR) of infants that developed late AKI (620; 550–740) and late severe AKI (580; 530–705) were lower than infants without AKI (730; 607–930) (Table 1). Other demographic data by late AKI status are shown in Table 1 - no other significant differences between groups were found. Regression analyses adjusted for gestational age given differences between comparison groups. Analyses did not additionally adjust for birth weight given collinearity with gestational age.

**Table 1:** Demographic characteristics by AKI status.

	No AKI (*N* = 177)	Any AKI (*N* = 39)	*p*-value^a^	Stage 2/3 AKI (*N* = 27)	*p*-value^b^
Gestational age (weeks), median (IQR)	25.9 (24.3–27.4)	24.1 (23.1–26.1)	<0.001	23.9 (23.1–25.9)	<0.001
<24 weeks	28 (16)	19 (49)	<0.001	14 (52)	<0.001
24–25 6/7 weeks	62 (35)	10 (26)	0.64	7 (26)	0.38
26–28 6/7 weeks	87 (49)	10 (26)	0.008	6 (22)	0.11
Birth weight (g), median (IQR)	730 (607–930)	620 (550–740)	0.006	580 (530–705)	0.001
Multiple gestation, *n* (%)	35 (20)	8 (21)	0.92	5 (19)	0.85
Male sex, *n* (%)	95 (54)	22 (56)	0.76	17 (63)	0.33
White race, *n* (%)	73 (41)	10 (26)	0.07	7 (26)	0.15
Antenatal corticosteroids, *n* (%)	162 (92)	36 (92)	0.87	24 (89)	0.58
Caesarean section, *n* (%)	120 (68)	25 (64)	0.66	18 (67)	0.96
Histologic chorioamnionitis, *n* (%)	86 (49)	19 (49)	0.99	12 (44)	0.64
Small for gestational age, *n* (%)	29 (16)	5 (13)	0.58	4 (15)	0.89
Prolonged rupture of membranes, *n* (%)	42 (24)	9 (24)	0.97	6 (23)	0.91

^a^No AKI vs. Any AKI.

^b^No AKI and Stage 1 AKI v Stage 2 and 3 AKI.

### Clinical outcomes

Clinical outcomes by late AKI strata are shown in Table 2. When considered as a binary covariate, a similar proportion of infants with late AKI (28%; *p* = 0.83) and late severe AKI (30%; *p* = 1.00) had a PDA compared to infants without AKI (30%). Similarly, the proportion of infants with late AKI (26%; *p* = 0.74) and late severe AKI (26%; *p* = 0.76) that had a HPDA were comparable to infants without AKI (23%). Infants with late AKI and late severe AKI had more severe grades of BPD. Of the infants without late AKI, 70% had grade 1 BPD, 23% had grade 2 BPD, and 7% had grade 3 BPD. Of the infants with late AKI, 36% had grade 1 BPD, 21% had grade 2 BPD, and 44% had grade 3 BPD (*p* < 0.001). For infants with late severe AKIs, 30% had grade 1 BPD, 22% had grade 2 BPD, and 48% had grade 3 BPD (*p* < 0.001) (Table 2). Of the 216 neonates in the cohort, 28 died. The mortality rate in infants with any late AKI was higher than in infants with no AKI (36% vs 6%; *p* < 0.001). The mortality rate was also higher in infants with late severe AKI (48%; *p* < 0.001) compared to infants without late AKI or stage 1 AKI (Table 2).

**Table 2:** Clinical outcomes by AKI status.

	No AKI (*N* = 177)	Any AKI (*N* = 39)	*P* value^a^	Stage 2/3 AKI (*N* = 27)	*P* value^b^
Bronchopulmonary dysplasia					
Grade 1	124 (70)	14 (36)	<0.001	8 (30)	<0.001
Grade 2	40 (23)	8 (21)		6 (22)	
Grade 3	13 (7)	17 (44)		13 (48)	
Patent ductus arteriosus	53 (30)	11 (28)	0.83	8 (30)	1.00
Moderate to large PDA	41 (23)	10 (26)	0.74	7 (26)	0.76
Pharmacologic treatment of PDA	48 (27)	13 (33)	0.44	10 (37)	0.28
Grade 3-4 intracranial hemorrhage	21 (12)	2 (5)	0.22	0 (0)	0.06
Early onset sepsis	2 (1)	2 (5)	0.09	1 (4)	0.45
Late onset sepsis	28 (16)	11 (28)	0.07	9 (33)	0.03
Severe retinopathy of prematurity	36 (20)	7 (18)	0.74	5 (19)	0.85
Necrotizing enterocolitis stage ≥ 2	17 (10)	8 (21)	0.05	8 (30)	0.002
Pulmonary hypertension	60 (34)	22 (56)	0.009	15 (56)	0.04
Death	11 (6)	14 (36)	<0.001	13 (48)	<0.001

^a^No AKI vs Any AKI.

^b^No AKI and stage 1 AKI v Stage 2 and 3 AKI.

### Patent ductus arteriosus exposure and AKI

There were 65 infants found to have a PDA on echocardiography after 28 postnatal days. Infants with a PDA had an odds ratio (OR) of 0.92 (95% CI 0.43–1.98) for late AKI, and an OR of 1.0 (95% Cl 0.41–2.42) for late severe AKI. When adjusted for gestational age, the ORs were 0.67 (95% Cl 0.30–1.51) for late AKI and 0.71 (95% Cl 0.28–1.81) for late severe AKI.

In evaluating strata of PDA exposure duration, there were no durations that increased the odds for late AKI: 4–7w (OR 0.74; 95% Cl 0.24–2.30), 8–11w (OR 0.32; 95% Cl 0.04–2.51), ≥12w (OR 1.77; 95% Cl 0.63–4.97). Similarly, there were no identified strata of PDA exposure duration that increased the odds for late severe AKI: 4–7w (OR 0.26; 95% CI 0.03–2.02), 8–11w (OR 0.50 (0.06–4.02), >12w (OR 2.80; 95% CI 0.97–8.10) (Table 3). These findings remained nonsignificant after adjustment for gestational age.

**Table 3:** Association of acute kidney injury with patent ductus arteriosus.

	No AKI (*n* = 177)	AKI (*n* = 39)	Crude OR (95% CI)	Adjusted OR^a^ (95% CI)
PDA duration				
No PDA	124 (70)	28 (72)	REF	REF
Any PDA	53 (30)	11 (10)	0.92 (0.43–1.98)	0.67 (0.30–1.51)
4–7w	24 (14)	4 (3)	0.74 (0.24–2.30)	0.50 (0.15–1.64)
8–11w	14 (8)	1 (15)	0.32 (0.04–2.51)	0.57 (0.43–0.75)
≥12w	15 (8)	6 (28)	1.77 (0.63–4.97)	1.14 (0.37–3.49)
HPDA duration				
No HPDA	136 (77)	29 (74)	REF	REF
Any HPDA	41 (23)	10 (26)	1.26 (0.58–2.75)	0.86 (0.38–1.97)
4–7w	21 (12)	4 (10)	0.67 (0.19–2.40)	0.39 (0.10–1.46)
8–11w	9 (5)	0 (0)	0.52 (0.06–4.27)	0.59 (0.07–5.27)
≥12w	11 (6)	6 (15)	2.56 (0.88–7.48)	1.49 (0.47–4.75)

^a^Adjusted for gestational age at birth.

### Hemodynamically significant patent ductus arteriosus exposure and AKI

Of the 64 infants with a PDA, 51 were deemed hemodynamically significant. Infants with a HPDA had an OR of 1.26 (95% Cl 0.58–2.75) for late AKI and an OR of 1.35 (95% Cl 0.55–3.29) for late severe AKI. When adjusted for gestational age the ORs were 0.86 (95% Cl 0.38–1.97) for late AKI and 0.89 (95% Cl 0.35–2.28) for late severe AKI.

There were no strata of HPDA duration that increased risk for late AKI: 4–7w (OR 0.67; 95% CI 0.19–2.40), 8–11w (OR 0.52; 95% CI 0.06–4.27), ≥12w (OR 2.56; 95% CI 0.88–7.48). These findings remained nonsignificant after adjustment for gestational age. For late severe AKI infants exposed to a HPDA for ≥ 12 weeks had an increased odds for late severe AKI (OR 3.96; 95% CI 1.32–11.87). When adjusting for gestational age, the association was not significant (aOR of 2.37; 95% CI 0.72–7.78). Shorter durations of HPDA exposure did not show a significant risk for late severe AKI wither by unadjusted or adjusted analyses (Table 4).

**Table 4:** Association of severe acute kidney injury with patent ductus arteriosus.

	No severe AKI (*n* = 189)	Severe AKI (*n* = 27)	Crude OR (95% CI)	Adjusted OR^a^ (95% CI)
PDA duration				
No PDA	133 (70)	19 (70)	REF	REF
Any PDA	56 (30)	8 (30)	1.0 (0.41–2.42)	0.71 (0.28–1.81)
4–7w	27 (14)	1 (4)	0.26 (0.03–2.02)	0.16 (0.02–1.32)
8–11w	14 (7)	1 (4)	0.50 (0.06–4.02)	0.53 (0.06–4.56)
≥12w	15 (8)	6 (22)	2.80 (0.97–8.10)	1.86 (0.59–5.91)
HPDA duration				
No HPDA	145 (77)	20 (74)	REF	REF
Any HPDA	44 (23)	7 (26)	1.35 (0.55–3.29)	0.89 (0.35–2.28)
4–7w	24 (13)	1 (4)	NS	NS
8–11w	9 (5)	0 (0)	0.81 (0.10–6.70)	0.97 (0.11–8.88)
≥12w	11 (6)	6 (22)	3.96 (1.32–11.87)^b^	2.37 (0.72–7.78)

^a^Adjusted for gestational age at birth.

^b^*p* < 0.05.

## Discussion

In this investigation infants with ≥12 weeks of HPDA exposure had an increased risk for late severe AKIs. However, given the known collinearity of PDA duration and prematurity, we adjusted for gestational age after which this association was no longer significant. When considering all PDAs and durations of exposure, there was no increased risk for any or severe late AKI. Stronger associations were identified between late AKI and the morbidities of BPD, NEC, and pulmonary hypertension as well as mortality.

This analysis was motivated by prior epidemiologic studies [9] reporting a decline in PDA treatment, which may present a longitudinal risk for late AKI in preterm infants. While available evidence supports the approach of therapeutic abstention [15], there is limited epidemiologic data analyzing the risk of PDA duration on the risk of late AKI and AKI severity. Herein, we describe duration of PDA exposure and the association of late onset AKI in extremely preterm infants. While there was no significant risk between prolonged PDA exposure and late AKI, there was a trend for a higher odds of late AKI with longer PDA exposures. However, observed differences in other important morbidities of prematurity by AKI status suggest residual confounders constraining associations between PDA exposure and late AKI risk.

Characteristics associated with late AKI risk, including shunt hemodynamics, demographic characteristics and comorbidities, are consistent with prior research. A hemodynamically significant PDA impairs systemic perfusion by diverting blood to the pulmonary circulation, diminishing renal blood flow and other end-organ perfusion. Prolonged exposure further compromises cardiac function, exacerbating low cardiac output and renal injury [3, 16, 17]. Lower gestational ages and birth weights correlated with more AKI and severe AKI [18]. There was also a correlation among infants who developed AKI and were also diagnosed with NEC, BPD, and pulmonary hypertension. The hemodynamic effects of such morbidities may independently increase infants’ risk for AKI. Finally, there was an association of death and late AKI, as established from the AWAKEN trial, where mortality of infants with AKI was much higher than infants that did not develop AKI.

Prior randomized trials in extremely preterm infants undergoing HPDA closure versus expectant management [5, 6] have not reported AKI or late onset AKI as an outcome. Duration of HPDA exposure in these trials was typically less than 4 weeks as this was the postnatal age of PDA closure. In studies evaluating AKI risk by HPDA exposure, some studies show no correlation [19] whereas others show significant risk of AKI [4, 20]. The impact of PDA exposure duration and outcomes for extremely preterm infants has been less thoroughly evaluated. Reports have suggested that preterm infants who are exposed to HPDAs for longer periods of time (>4weeks) have increased risks of BPD and neurologic changes [10, 21, 22]. It is also well established that exposure to large PDAs in children have a significant effect on volume overloading of the heart [3, 23].

This investigation has several strengths. Our center’s use of monthly, longitudinal echocardiography enabled an analysis of PDA exposure duration and late AKI risk. This systematic use of screening echocardiograms also limits the introduction of selection bias, although the inclusion of only infants on respiratory support on postnatal day 28 limits generalizability to the entire extremely preterm population. We also studied a large population of extremely preterm infants providing the ability to associate PDA exposures with AKI outcomes. Limitations include data being limited to a single center and the retrospective, observational study design which cannot claim causal links between exposures and outcomes. Characterization of a PDA as hemodynamically significant may also vary by operator and patient size. Moreover, the specific criteria by which a PDA was deemed hemodynamically significant were not collected or reported. In addition, study design limited our ability to investigate pharmacotherapy exposures (e.g. nephrotoxic antibiotics, diuretics, and corticosteroids) were not evaluated. Lastly, the causes of AKI are multifactorial, limiting the ability to precisely define the relationship between PDA duration and AKI risk.

## Conclusion

While there are many known risk factors associated with AKI in extremely preterm infants, evidence from our investigation demonstrates a potential association between longer exposures to HPDAs and risk for late severe AKIs. In addition to conferring further validation, additional, multicenter studies may be capable of analyzing a greater number of infants with a HPDA and relating the duration of this exposure to risk for late AKI. Future randomized trials evaluating PDA management should report the outcomes of any and severe AKI. With more extremely preterm infants being exposed to a HPDA for longer periods of time [24], further investigation of whether certain PDA exposure durations increase infants’ risk for morbidity are needed.

## Data Availability

The datasets generated and/or analyzed during the current study are not publicly available due to restrictions related to the use of identifiable private information, but they are available from the corresponding author on reasonable request and with appropriate institutional approvals.
